# Development and validation of Gaucher disease type 1 (GD1)-specific patient-reported outcome measures (PROMs) for clinical monitoring and for clinical trials

**DOI:** 10.1186/s13023-021-02163-y

**Published:** 2022-01-06

**Authors:** Deborah Elstein, Nadia Belmatoug, Patrick Deegan, Özlem Göker-Alpan, Derralynn A. Hughes, Ida Vanessa D. Schwartz, Neal Weinreb, Nicola Bonner, Charlotte Panter, Donna Fountain, Andrew Lenny, Louise Longworth, Rachael Miller, Koonal Shah, Jörn Schenk, Rohini Sen, Ari Zimran

**Affiliations:** 1 Takeda Pharmaceuticals International AG, Zurich, Switzerland; 2 Assistance-Publique Hôpitaux de Paris Nord, Université de Paris, Paris, France; 3 Lysosomal Disorders Unit, Cambridge University Hospitals, Cambridge, UK; 4 Lysosomal Disorders Unit and Center for Clinical Trials, O&O Alpan LLC, Fairfax, VA USA; 5 Royal Free London NHS Foundation Trust, London, UK; 6 Medical Genetics Service - HCPA, Genetics Department, UFRGS, Porte Alegre, Brazil; 7 University of Miami Miller School of Medicine, Miami, FL USA; 8 Adelphi Values, Bollington, UK; 9 PHMR, London, UK; 10 Takeda Pharmaceuticals Company Ltd, Cambridge, MA USA; 11 Gaucher Unit, Shaare Zedek Medical Center, Jerusalem, Israel; 12 Hebrew University-Hadassah Medical School, Jerusalem, Israel

**Keywords:** Patient-reported outcomes, PROM, Gaucher disease, Lysosomal storage disorder, Questionnaire, Content validation, Psychometric validation

## Abstract

**Background:**

Disease-specific patient-reported outcome measures (PROMs) are fundamental to understanding the impact on, and expectations of, patients with genetic disorders, and can facilitate constructive and educated conversations about treatments and outcomes. However, generic PROMs may fail to capture disease-specific concerns. Here we report the development and validation of a Gaucher disease (GD)-specific PROM for patients with type 1 Gaucher disease (GD1) a lysosomal storage disorder characterized by hepatosplenomegaly, thrombocytopenia, anemia, bruising, bone disease, and fatigue.

**Results and discussion:**

The questionnaire was initially developed with input from 85 patients or parents of patients with GD1 or GD3 in Israel. Owing to few participating patients with GD3, content validity was assessed for patients with GD1 only. Content validity of the revised questionnaire was assessed in 33 patients in the US, France, and Israel according to US Food and Drug Administration standards, with input from a panel of six GD experts and one patient advocate representative. Concept elicitation interviews explored patient experience of symptoms and treatments, and a cognitive debriefing exercise explored patients’ understanding and relevance of instructions, items, response scales, and recall period. Two versions of the questionnaire were subsequently developed: a 24-item version for routine monitoring in clinical practice (rmGD1-PROM), and a 17-item version for use in clinical trials (ctGD1-PROM). Psychometric validation of the ctGD1-PROM was assessed in 46 adult patients with GD1 and re-administered two weeks later to examine test–retest reliability. Findings from the psychometric validation study revealed excellent internal consistency and strong evidence of convergent validity of the ctGD1-PROM based on correlations with the 36-item Short Form Health Survey. Most items were found to show moderate, good, or excellent test–retest reliability.

**Conclusions:**

Development of the ctGD1-PROM represents an important step forward for researchers measuring the impact of GD and its respective treatment.

**Supplementary Information:**

The online version contains supplementary material available at 10.1186/s13023-021-02163-y.

## Introduction

Gaucher disease (GD) is an autosomal recessive disorder characterized by a deficiency in the lysosomal enzyme acid β-glucosidase (GCase), caused by pathogenic variation in the *GBA1* gene [1]. The most frequent form, GD type 1 (GD1), is associated with enlargement of the spleen and liver, the presence of thrombocytopenia and anemia, and bone disease that may include osteoporosis with susceptibility to fragility fractures, osteonecrosis with joint collapse, and acute as well as chronic bone pain. GD1 is not associated with neurologic manifestations, although there is a well-documented association with Parkinson’s disease [2]. The phenotypic variation is broad, encompassing individuals who remain mildly affected or asymptomatic through to elderly age as well as others in whom manifestations become evident from childhood to early adulthood [1]. Worldwide, thousands of patients with GD1 over the last 25 years have benefited from intravenous treatment with pharmacologic recombinant GCases (enzyme replacement therapy; ERT) and more recently from oral, small-molecule inhibitors of glucosylceramide synthase (substrate reduction therapy). Neuronopathic variants, GD2 and GD3, are characterised by neurologic manifestations, in addition to the spectrum of signs and symptoms found in non-neuronopathic GD1 [1]. Patients with GD2 and GD3 may benefit systemically from ERT, although neurologic deterioration is unaffected [3–6]. At the more severe end of the phenotypic spectrum, GD2 is characterized by devastating central nervous system and systemic involvement manifesting either at birth or in infancy, and affected infants rarely live for >2–3 years [7, 8].

The importance of individualized patient-centric monitoring is now widely recognized, both with regard to individual patient management and for informing commissioning of healthcare services, and the US Food and Drug Administration (FDA) has issued guidance as to how these measures should be incorporated in clinical trial design [9]. Generic measures of health-related quality of life (HRQoL), including the 36-item Short Form Health Survey (SF-36), the EuroQoL-5 Dimension (EQ-5D) [10], and the Lansky play performance scale for children [11], have been used in rare disease clinical trials and in post-approval surveillance studies, including those for GD1 [12–20]. However, because of their generality, these HRQoL instruments may miss important nuances of the disease by failing to capture disease-specific patient-reported outcome measures (PROMs). Further, the overlay of psychological, social, and societal concerns and stressors is unique to patients with chronic but rare disorders that are often erratically progressive and impact lifestyle in a multi-factorial fashion [21]. These latter factors, too, need to be evaluated within the GD-specific spectrum of outcomes among treated as well as untreated patients.

In this evolving environment, PROMs that convincingly provide evidence of significant improvements in HRQoL with consequent individual and societal benefits will be crucial to treatment-approval processes across the board, but especially for rare disorders such as GD, for which sustained, effective therapy should result in healthy and “normal” life expectancy. At a population level, disease-specific PROMs can inform healthcare commissioning, while at an individual level, a disease-specific PROM can facilitate patient/physician dialogue based on HRQoL responses. This might more clearly indicate the individual patient’s current mindset and expectations, enlighten clinical management, and in turn, motivate patients to be active participants in their care.

A GD-specific PROM (the GD1-PROM) was originally developed and circulated by Deborah Elstein to afford greater insight into the condition of the HRQoL of patients with GD1. Further work resulted in development of two versions of the GD1-PROM: a routine monitoring version for clinical practice, and a version for use in clinical trials. Here we describe the content and psychometric validation of the GD1-PROM, as well as required measurement properties per FDA guidance [9].

## Initial development of the questionnaire

### Study design

The initial version of the questionnaire was based on input from patients receiving treatment at the Shaare Zedek Medical Center, Jerusalem. It was designed to be comparable with the SF-36 questionnaire [22] for aspects relating to general HRQoL and additionally include original questions covering GD-specific aspects and orphan drug-specific aspects. The first draft of the questionnaire included 11 questions revised from the SF-36 questionnaire, nine originally developed GD-specific questions, three originally developed orphan drug-specific questions, and seven originally developed activities of daily living, symptoms, and psychosocial items. The questionnaire was drafted in English and Hebrew and additionally translated into Arabic from the English version by a native speaker (Rinad Nabulsi, MD).

### Patient input

**Fig. 1:**
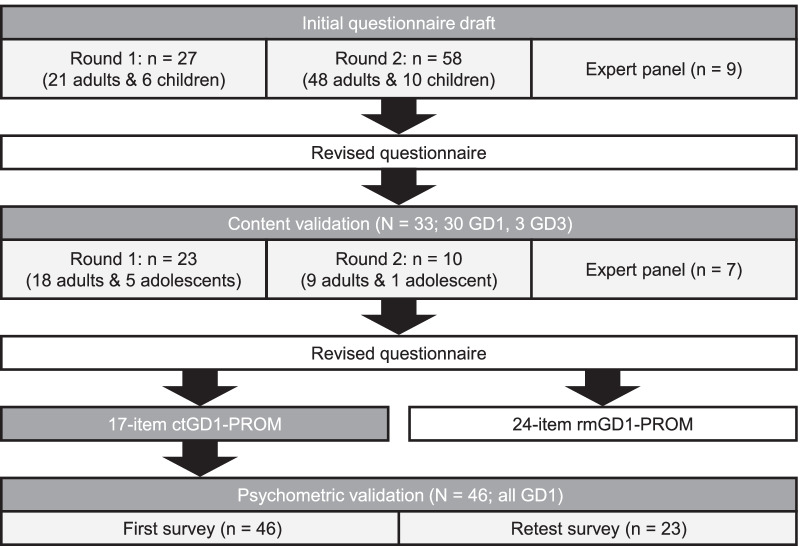
Overview of the development and validation of the ctGD1-PROM. *ctGD1-PROM* clinical trial (17-item) GD1-specific patient-reported outcome measure questionnaire, *GD1/3* Gaucher disease type 1/3, *rmGD1-PROM* routine monitoring (24-item) GD1-specific patient-reported outcome measure questionnaire

The initial version of the questionnaire was administered to 21 adult patients and six parents of pediatric patients aged < 12 years at the Gaucher Clinic at Shaare Zedek Medical Center, Jerusalem, under the directorship of Professor Zimran for routine follow-up (Fig. 1). Patients provided detailed feedback on the content and language used in version 1 of the questionnaire, which was used to inform revisions. A revised questionnaire (version 2) was next administered to 48 patients (82.8%) and 10 parents of patients (17.2%) from the same center, of whom 38 (65.5%) patients were receiving GD-specific therapy (Fig. 1). Most patients were administered the Hebrew version (86.2%), six (10.6%) patients received the Arabic version, and two (3.4%) patients received the English version.

### Specialist clinician input

A panel of experts provided input into a third version of the questionnaire, with no changes requested (Fig. 1). The panel comprised five clinicians with expertise in GD: Dr. Neal Weinreb, Dr. Özlem Göker-Alpan, Dr. Nadia Belmatoug, Professor Ida Vanessa D. Schwartz, and Dr. Patrick Deegan; two Canadian experts in PROMs: Professor Gordon Guyatt and Dr. Patricia Miller, both at McMaster University in Hamilton, Ontario, Canada; and two representatives of the European Gaucher Patients Alliance: Jeremy Manuel, OBE, and Tanya Collin-Histed. At this point, Shire (now Takeda) was given the rights to the PROM for validation and to make it freely available upon completion of that process.

## Content validation

### Study design

Following the development of the initial questionnaire, a content validation study was conducted to develop/adapt and assess the questionnaire to confirm its suitability for use in clinical practice as well as in clinical trials (Fig. 1). This was a cross-sectional, non-interventional, qualitative study involving two rounds of concept elicitation and cognitive debriefing interviews with adults and adolescents with GD1 or GD3. Each round of patient interviews was followed by input from a panel of six GD experts (N. Weinreb, Ö. Göker-Alpan, N Belmatoug, I.V. Schwartz, P. Deegan, and D. Elstein) and one patient advocate (Tanya Collin-Histed from the European Gaucher Alliance, now the International Gaucher Alliance) to review the clinical relevance of changes made to the questionnaire based on patient feedback. A semi-structured interview guide was used to guide the conduct of the interviews, which were carried out by trained interviewers in the local language of the interviewee (English, French, Arabic, or Hebrew). Eligible patients, literate and fluent in the language of the country where they were residing and with a physician-confirmed diagnosis of GD1 or GD3, were recruited from four specialist clinical sites in the US, France, and Israel. Participants provided written informed consent before the conduct of any study-related activities. This study was approved by an international and independent ethical review board.

The planned sample size was determined based on the principle of “concept saturation”. Concept saturation is commonly defined as the point at which no new and important concepts relevant to the research question are identified emerging from iterative rounds of interviews (i.e. collecting additional data will not likely add to the understanding of how participants perceive the concept of interest) [23, 24]. Past experience and evidence in the literature suggests that concept saturation can be achieved in as few as 12–15 individual interviews and that 99.3% of concepts typically emerge within 25 interviews [25]. As such, it was estimated that an overall minimum sample of 30 participants would be adequate to achieve saturation.

Qualitative analysis of transcripts was conducted using the computer-assisted qualitative data analysis software program, ATLAS.ti.16. Transcripts were analyzed using thematic analysis methods, and participant quotes that pertained to the main research objectives were highlighted and assigned corresponding concept codes.

### Patients and recruitment

A total of 33 patients ≥ 12 years of age with GD1 or GD3 were recruited into the content validation study: 23 participated in round 1 of the qualitative interviews (18 adults and five adolescents) and 10 participated in round 2 (nine adults and one adolescent) (Fig. 1). Thirty patients had GD1; only three patients had GD3. Thirty were receiving treatment (26 with GD1 and three with GD3) and four were treatment naïve. Demographic characteristics were similar for rounds 1 and 2 (Table 1).

**Table 1:** Content validation: demographic characteristics of the study population

Characteristic	Round 1 n = 23	Round 2 n = 10	Total n = 33
Mean (range) age, years	35.7 (13–67)	50.9 (15–73)	40.3 (13–73)
Sex, n (%)			
Female	14 (60.9)	7 (70.0)	21 (63.6)
Ethnicity, n (%)*			
White	11 (47.8)	7 (70.0)	18 (54.5)
Ashkenazi Jewish	11 (47.8)	3 (30.0)	14 (42.4)
Other	4 (17.4)	0	4 (12.1)
Highest educational level (adults only), n (%)			
Some high school	1 (4.3)	0	1 (3.0)
High school diploma or General Educational Development (GED)	3 (13.0)	1 (10.0)	4 (12.1)
Some years of college	3 (13.0)	1 (10.0)	4 (12.1)
Certificate program	1 (4.3)	0	1 (3.0)
University/college	3 (13.0)	2 (20.0)	5 (15.2)
Graduate or professional degree	5 (21.7)	4 (40.0)	9 (27.3)
Other	1 (4.3)	1 (10.0)	2 (6.1)
N/A or missing	6 (26.1)	0	7 (21.2)
Mean (range) duration of disease, years	26.2 (1–60)	35.0 (14–63)	26.8 (1–63)
Disease severity (as rated by the recruiting clinician), n (%)			
Very mild	1 (4.3)	0	1 (3.0)
Mild	5 (21.7)	2 (20.0)	7 (21.2)
Moderate	14 (60.9)	4 (40.0)	18 (54.5)
Severe	3 (13.0)	3 (30.0)	6 (18.2)
Very severe	0	1 (10.0)	1 (3.0)

*Multiple answers given

### Concept elicitation interviews

Recruited participants took part in a 90-min combined concept elicitation and cognitive debriefing interview. The focus of this portion of the interview was to establish how GD affects patients with respect to their symptoms, impacts on functioning/HRQoL, and treatment experience. The concepts elicited were used to develop a conceptual model for GD, which detailed the overall patient experience of GD following the theory of the Wilson and Cleary model [26]. The model was then used to assess the conceptual coverage of the questionnaire (i.e. the proportion of concepts covered by the questionnaire) and inform any modifications.

**Fig. 2:**
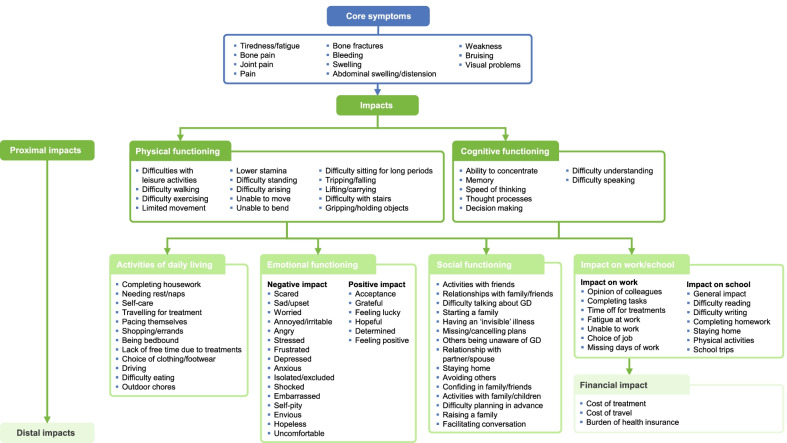
Content validation: conceptual model. *GD* Gaucher disease

**Fig. 3:**
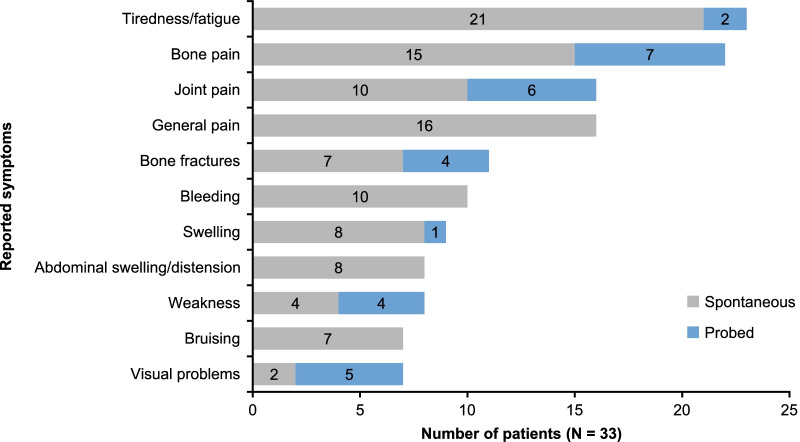
Content validation: key symptoms of GD reported by patients. *GD* Gaucher disease

**Fig. 4:**
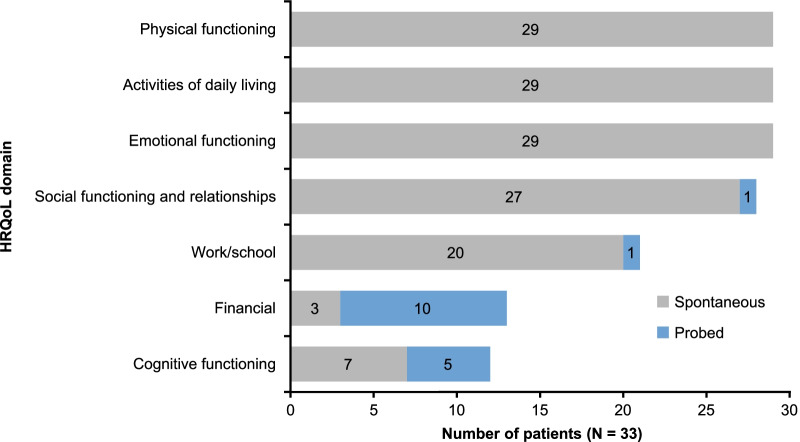
Content validation: impact on HRQoL domains reported by patients. *HRQoL* health-related quality of life

Concept elicitation interviews resulted in 11 core symptoms of GD and seven core impact categories being reported by patients (Fig. 2). The most reported were tiredness/fatigue (n = 23; 69.7%), bone pain (n = 22; 66.7%), joint pain (n = 16; 48.5%), general pain (n = 16; 48.5%), and bone fractures (n = 11; 33.3%) (Fig. 3). All patients described at least one way in which they had been affected by their GD. Most patients spontaneously (without probing) described an impact on physical functioning, activities of daily living, and emotional functioning HRQoL domains (n = 29 for each; 87.9%) (Fig. 4). Few (three [9%]) patients spontaneously described a financial impact, with an additional 10 patients reporting this impact upon probing (Fig. 4).

Evaluation of concept elicitation interviews was completed at the symptom level by dividing transcripts into three sets of 10 interview transcripts. For adult patients with GD1, most symptom concepts emerged in the first two sets of interviews, with only kidney pain and seizures emerging in the final set of interviews. The rare nature of GD3 and the low number of adolescent participants led to challenges in recruiting adequate sample sizes for comparative analyses between GD1 and GD3, and between adults and adolescents.

### Cognitive debriefing interviews

The aim of the cognitive debriefing component was to ask patients about their understanding of instructions and item wording, and about the relevance and comprehensiveness of the items included. The cognitive debriefing section also assessed the appropriateness of the response options and recall period for all items.

In round 1 of the cognitive debriefing interviews, 23 patients were debriefed on the 30-item questionnaire. Part 1 (questions 1–23) employed a “yes/no/not relevant” response scale and part 2 (questions 24–30) employed a 0–10 numeric rating scale. Many items were well understood by patients; however, some items appeared to lack conceptual relevance. The findings were discussed with the expert panel and carefully reviewed against regulatory criteria on the development of PROMs. In line with the FDA PROM guidance [9], key changes after the first round of interviews included: the addition of a recall period of “over the past month” to the majority of items in part 1 and “over the past week” to items in part 2; modification of part 1 questions to employ a 0–4 verbal response scale (from “none of the time” to “all of the time” [13 questions] or from “strongly agree” to “strongly disagree” [2 questions]); removal of five items from part 1 owing to lack of conceptual relevance; and inclusion of six additional items to part 2, based on the concept elicitation findings (abdominal swelling, physical weakness, joint swelling, worry, memory, and mobility).

In round 2 of the cognitive debriefing interviews, 10 patients were debriefed on the revised 31-item version of the questionnaire. Most items were well understood by all patients and considered relevant by ≥ 50% of the sample. However, eight items were not understood by one adult participant each. The participant-level findings also indicated that while half of the participants understood all items, the other half had difficulty with only one or two isolated items. Further modifications made to the questionnaire included the removal of seven items, including three items from part 1 and four items from part 2, owing to a lack of conceptual relevance. Addition of a “not applicable or prefer not to say” response was made to 13 of the 5-point (0–4) verbal response scale questions in part 1. All response anchors in part 2 were reversed so that a higher score indicates a higher level of impact, as this made the most sense to participants.

After concept elicitation and cognitive debriefing interviews, the questionnaire was modified to consist of 15 questions with a 6-point verbal response scale (part 1), and nine questions using a 0–10 numeric rating scale (part 2), resulting in a 24-item questionnaire that is relevant and easily understood for patients with GD of varying levels of educational ability (Table 2).

**Table 2:** Overview of the rmGD1-PROM and ctGD1-PROM questions and structure

Part	No. of questions	Topics	Scale
ctGD1-PROM and rmGD1-PROM			
1A	4	Restriction of activities, education, and work	6-point verbal response scale
	1	Concern about emotional burden	
1B	2	Context of GD concerns relating to general health and medication	5-point verbal response scale
	1	Context of GD concerns relating to other medical concerns	
2	1	Dependence on others	0–10 numeric rating scale
	6	Presence/severity of symptoms	
	1	Satisfaction with treatment	
	1	Concern about the future	
rmGD1-PROM only			
	6	Concern about comorbidities, disease burden, and cost	6-point verbal response scale
	1	Context of GD concerns relating to other medical concerns	5-point verbal response scale

*ctGD1-PROM* clinical trials (17-item) Gaucher disease type 1-specific patient-reported outcome measure questionnaire, *GD* Gaucher disease, *rmGD1-PROM* routine monitoring (24-item) GD1-specific patient-reported outcome measure questionnaire

## Psychometric validation

Owing to an expectation that some items, although considered clinically relevant by GD experts, would not be expected to change over the course of a clinical trial, coupled with advice from the UK National Health Service (NHS) Research Ethics Committee that some items may be distressing for patients, the decision was made that the full 24-item version of the questionnaire would be pursued for routine monitoring in clinical practice (rmGD1-PROM; Additional file 1), and a shorter, 17-item version would undergo psychometric validation for use in clinical trials (ctGD1-PROM). Psychometric analyses were undertaken to establish the measurement properties of the 17-item ctGD1-PROM, which includes eight questions from part 1 and all nine questions from part 2 of the full-length, 24-item rmGD1-PROM (Fig. 1, Table 2).

### Study design

Psychometric validation, including validity and reliability, was assessed by means of a patient survey study administered to patients aged ≥ 18 years with confirmed GD1 who were receiving treatment at the Royal Free London NHS Foundation Trust, London, UK, under the care of Dr. Derralynn Hughes. Patients received by post an invitation letter, information sheet, and consent form, along with the main survey and a pre-paid envelope for its return. A reminder letter was sent to participants 4 weeks later. The survey comprised the GD-PROM, the SF-36, and questions on socio-demographics (including age, sex, ethnicity, and occupational status) and disease history (including self-assessment of health status, time since initial diagnosis, and date of last visit to the specialist center). The survey was re-administered two weeks after the initial administration to examine test–retest reliability. Responses were entered into an Excel database designed specifically for the study by two analysts independently, with a third senior analyst comparing the two sets of data for discrepancies, referring to the paper questionnaires to resolve any differences.

In addition to survey completion, data on disease severity extracted from the Gaucher Outcomes Survey (GOS) registry (an ongoing registry for patients with GD, in which participating patients were enrolled, irrespective of treatment status or treatment type (NCT03291223) [27]), were assessed using the GD1 disease severity scoring system (GD1-DS3), described by Weinreb et al., 2010 [28]. Data required for the completion of the GD1-DS3 were evaluated by a clinician based at the Royal Free London NHS Foundation Trust, to produce the summary score for each patient.

Consent to participate in the study was obtained from participants at the same time as completion of the questionnaire. The study protocol and related documents were approved by the NHS Research Ethics Committee before initiation of any study procedures. Background characteristics were examined using descriptive statistics, and sensitivity analyses were used to assess the impact on psychometric analyses of excluding patients who completed the questionnaires >24 months after their last GD-related health appointment.

### Patient sample

Fifty patients completed the survey. Of these, three did not provide consent to participate in the study so were excluded from the analysis. One further respondent did not complete the ctGD1-PROM but completed the rest of the survey, so was also excluded from the analysis. In total, 46 initial ctGD1-PROM surveys and 23 follow-up surveys were analyzed (Fig. 1). Most patients were diagnosed with GD > 20 years ago, were White, employed, and nearly half had a GD1-DS3 score of < 3 (mild disease) (Table 3).

**Table 3:** Psychometric validation: demographics characteristics of the study population

	Main sample (N = 46)		Test–retest sample (n = 23)	
Characteristic	n	%	n	%
Age, years				
25**–**34	9	19.6	4	17.4
35**–**44	13	28.3	6	26.1
45**–**54	3	6.5	1	4.3
55**–**64	12	26.1	5	21.7
65**–**74	5	10.9	5	21.7
≥75	4	8.7	2	8.7
Gender				
Male	23	50.0	10	43.5
Female	22	47.8	12	52.2
Missing	1	2.2	1	4.3
Ethnicity				
White	45	97.8	22	95.7
Other	1	2.2	1	4.3
Occupational status				
Employed part-time	8	17.4	3	13.0
Employed full-time	14	30.4	6	26.1
Self-employed	4	8.7	1	4.3
Unemployed	5	10.9	2	8.7
Retired	13	28.3	9	39.1
Missing	2	4.3	2	8.7
Time since diagnosis				
<1 year	2	4.3	0	0.0
1**–**4 years	3	6.5	1	4.3
5**–**9 years	5	10.9	4	17.4
10**–**19 years	6	13.0	2	8.7
≥20 years	29	63.0	16	69.6
Missing	1	2.2	0	0.0
Current health				
Very good	7	15.2	4	17.4
Good	20	43.5	9	39.1
Fair	13	28.3	6	26.1
Bad	4	8.7	3	13.0
Very bad	2	4.3	1	4.3
Gaucher disease severity state				
Mild (GD1-DS3 score < 3.00)	21	45.7	12	52.2
Moderate (GD1-DS3 score 3.00**–**5.99)	7	15.2	2	8.7
Marked (GD1-DS3 score 6.00**–**8.99)	11	23.9	3	13.0
Severe (GD1-DS3 score 9.00**–**19.00)	7	15.2	6	26.1

*GD1-DS3* Gaucher disease type 1- disease severity scoring system (three domains)

### Validity and reliability of the ctGD1-PROM

Initial results showed strong evidence of convergent validity, based on correlations between overall and item-level ctGD1-PROM scores and the physical and mental component summary scores of the SF-36. Overall correlation coefficients were > 0.7, *p* < 0.001, and most item-level correlation coefficients were > 0.5, *p* < 0.05 (Table 4).

**Table 4:** Psychometric validation: correlations between the ctGD1-PROM items and SF-36 PCS and MCS scores

Items	Correlation with PCS			Correlation with MCS		
	Coefficient	(*p* < 0.05)	(*p* < 0.1)	Coefficient	(*p* < 0.05)	(*p* < 0.1)
GD Education/job	0.739	✓	✓	0.792	✓	✓
GD Friends	0.594	✓	✓	0.722	✓	✓
GD Intimate	0.633	✓	✓	0.702	✓	✓
GD Hobbies leisure	0.684	✓	✓	0.724	✓	✓
GD Emotional burden	0.616	✓	✓	0.742	✓	✓
GD General-specific med	0.205	x	x	0.383	✓	✓
GD Concerns Gaucher	0.139	x	x	0.023	x	x
GD Current med. concerns	0.172	x	x	0.358	✓	✓
GD Dependent	0.715	✓	✓	0.274	x	✓
GD Abdomen	0.478	✓	✓	0.415	✓	✓
GD Fatigued	0.680	✓	✓	0.609	✓	✓
GD Physical weakness	0.750	✓	✓	0.615	✓	✓
GD Bone pain	0.787	✓	✓	0.594	✓	✓
GD Depressed	0.514	✓	✓	0.808	✓	✓
GD Worried	0.566	✓	✓	0.819	✓	✓
GD Future	0.567	✓	✓	0.647	✓	✓
GD Satisfied	0.656	✓	✓	0.691	✓	✓

*GD* Gaucher disease, *MCS* mental component score, *PCS* physical component score, *SF-36* 36-item Short Form Health Survey

In terms of reliability, the overall Cronbach’s alpha for the ctGD1-PROM was 0.928, indicating excellent internal consistency (Table 5). The reproducibility of the ctGD1-PROM was examined across repeat administrations to determine the test–retest reliability of the measure based on intraclass correlation coefficients (ICCs). Most items, with two exceptions (GD Depressed and GD Satisfied), were found to show moderate, good, or excellent test–retest reliability (ICC ≥ 0.5), although the sample size was small (Table 6).

**Table 5:** Psychometric validation: internal consistency reliability statistics for the ctGD1-PROM

Item	Item-test correlation	Item-rest correlation	Inter-item correlation	Cronbach’s alpha
GD Education/job	0.658	0.605	0.434	0.925
GD Friends	0.783	0.745	0.422	0.921
GD Intimate	0.576	0.514	0.442	0.927
GD Hobbies leisure	0.741	0.696	0.426	0.922
GD Emotional burden	0.848	0.821	0.415	0.919
GD General-specific med	0.356	0.276	0.464	0.933
GD Concerns Gaucher	0.208	0.118	0.476	0.936
GD Current med. concerns	0.364	0.286	0.463	0.932
GD Dependent	0.594	0.533	0.439	0.926
GD Abdomen	0.616	0.553	0.438	0.926
GD Fatigued	0.802	0.767	0.420	0.921
GD Physical weakness	0.868	0.844	0.413	0.919
GD Bone pain	0.868	0.843	0.413	0.919
GD Depressed	0.834	0.805	0.417	0.920
GD Worried	0.870	0.846	0.413	0.919
GD Future	0.792	0.757	0.421	0.921
GD Satisfied	0.826	0.795	0.417	0.920
**Overall**			0.431	0.928

*ctGD1-PROM* clinical trials (17-item) Gaucher disease type 1-specific patient-reported outcome measure questionnaire, *GD* Gaucher disease

**Table 6:** Psychometric validation: test–retest intraclass correlations of the ctGD1-PROM (n = 23)

Item	Observations	ICC	95% confidence interval	
GD Education/job	22	0.509	0.118	0.763
GD Friends	23	0.682	0.384	0.851
GD Intimate	22	0.613	0.264	0.820
GD Hobbies leisure	23	0.757	0.508	0.889
GD Emotional burden	22	0.626	0.297	0.824
GD General-specific med	23	0.567	0.204	0.791
GD Concerns Gaucher	23	0.625	0.303	0.821
GD Current med. concerns	22	0.530	0.141	0.775
GD Dependent	22	0.500	0.107	0.757
GD Abdomen	22	0.974	0.939	0.989
GD Fatigued	22	0.816	0.606	0.919
GD Physical weakness	22	0.900	0.775	0.957
GD Bone pain	22	0.798	0.575	0.911
GD Depressed	22	0.482	0.075	0.749
GD Worried	22	0.512	0.123	0.764
GD Future	22	0.843	0.598	0.937
GD Satisfied	23	0.399	0.014	0.693
**Average**		0.893	0.750	0.954

*ctGD1-PROM* clinical trials (17-item) Gaucher disease type 1-specific patient-reported outcome measure questionnaire, *GD* Gaucher disease, *ICC* intraclass correlation coefficient

Known-groups validity was not demonstrated, indicating that the measure was unable to distinguish between severity groups based on the GD1-DS3. For the majority of items, patients in the moderate severity group had the highest mean response. Only one item (GD Bone Pain) showed increasing response with increasing severity. Using the analysis of variance (ANOVA) F-test, only one item (GD General-specific med) gave a *p* value < 0.10, indicating ability to distinguish between severity groups on this measure.

## Discussion

PROMs are now widely recognized as being crucial for assessment of the impact of disease and its treatment. However, there are few validated, disease-specific PROMs for rare diseases. Small sample sizes and heterogeneous study populations create substantial barriers to their development, with additional obstacles related to representative sampling, data collection, and statistical power. As a result, most rare diseases employ generic questionnaires for both clinical monitoring and clinical trials; however, these often fail to target the specific disease-related issues that patients experience [29, 30]. In the case of GD, the development and improvement of PROMs was flagged as a goal of a consensus panel consisting of the European Working Group on GD and patients with GD [31]. The development of this GD-specific PROM and its eventual wide availability are intended to afford greater insight into the condition of the individual patient as well as the status of patients over time, whether in the context of routine monitoring or a clinical trial [32].

The format of the proposed ctGD1-PROM is based on decades of experience with patients and personal involvement in clinical trials for GD. This cumulative expertise, supplemented with information from patient and disease registries, makes us comfortable in asserting that we have identified the issues that matter most to patients with GD1. We have also paid attention to how disease dynamics affect patients’ psychosocial health. GD is not only clinically heterogeneous at the time of diagnosis but also has a disease trajectory marked by periods of quiescence that may be unpredictably interrupted by complications and exacerbations. The effect of current treatments on later-life GD-related disorders, such as Parkinsonism, peripheral neuropathy, and malignancies (e.g. monoclonal gammopathy of undetermined significance/myeloma, other hematologic cancers, hepatocellular carcinoma), is unknown. The effects of this prognostic uncertainty need to be captured when assessing HRQoL. The ctGD1-PROM presented here is the first PROM for GD that documents these GD-specific patient concerns. This study therefore represents an important breakthrough in QoL research for this rare disease.

As GD is a rare disease, it was important not to impose too many sampling quotas that could restrict recruitment into the content validation study. Demographically, there was an adequate representation of males and females, and different education levels (important for cognitive debriefing). In line with literature that reports GD as particularly prevalent among Jews of Ashkenazi descent [33], almost half of the sample was Ashkenazi Jewish. Hispanic/Latino patients were under-represented in the sample, with only two recruited, and patients from Far Eastern populations that generally lack the N370S variant (c.1226A>G; p.Asn409Ser; now referred to as N409S) were not represented in the sample population at all.

The findings of qualitative interviews for content validation indicated that patients with GD experience a wide range of different disease manifestations that negatively impact their QoL. Signs and symptoms most commonly identified included tiredness or fatigue, bone pain, joint pain, pain (predominantly in the limbs, back, or stomach), bone fractures, bleeding, swelling (predominantly in the joints), abdominal swelling or distension, weakness, bruising, and visual problems, consistent with the previous findings [34].

Results of psychometric validation analyses show that the ctGD1-PROM performs reasonably well in terms of several key psychometric properties. Data completeness was acceptable, with the majority of respondents providing all the required data and no single ctGD1-PROM item accounting for more than two missing values. Strong evidence of convergent validity was found, based on correlations with two key SF-36 summary scores, and internal consistency was found to be excellent, with a very high Cronbach’s alpha coefficient for the overall questionnaire. Test–retest reproducibility was also found to be acceptable, with two items failing to show moderate reliability in this regard. However, neither the individual items nor the proposed overall questionnaire scores were able to discriminate well between the severity groups based on the GD1-DS3, the benchmark disease severity scoring system for GD1. This may be a reflection of the small sample size, the predominance of patients reporting their current health as good or very good, or a possible effect of weighting factors related to the construct of the GD1-DS3 total score, where patients may attribute a greater impact of certain items than the score allows. Further evaluation is required to assess the applicability of the ctGD1-PROM to patients with severe disease and/or not receiving treatment. The very high level of homogeneity between the items of the questionnaire, as shown by the magnitude of the Cronbach’s alpha coefficient, could indicate that some items are asking the same question, albeit in different ways. To further examine this argument, an exploratory factor analysis with a larger study sample is required. Consideration could also be given to the evaluation of the ctGD1-PROM in longitudinal studies to assess the responsiveness of the questionnaire in capturing changes in HRQoL over time. Qualitative research with patients to establish their perceptions of changes in their health as part of the longitudinal assessment could also be valuable for the assessment.

The part 1B items were found to behave differently from the rest of the items. While part 1A and part 2 items describe health and QoL problems and restrictions that respondents experience as a result of their GD, the three-question part 1B items focus on the impact of their medication or on the extent to which all of their medical concerns were GD related. Some of the psychometric analyses (e.g. internal consistency and convergent validity) show that these items do not perform as well as the other items. However, one of these three items—GD general-specific med—was the only one that appeared to be able to distinguish between known severity groups based on ANOVA F-testing.

There are some limitations to the study. It should be recognized that while the content validation study design provided considerable depth of insight and descriptions regarding the patient experience, caution should be employed when drawing conclusions. Adolescents and patients with GD3 were under-represented in the sample; therefore, it was not possible to draw any firm conclusions regarding any similarities or differences between the GD1/GD3 and adult/adolescent experience of GD. As a result, psychometric testing was undertaken only in adults with GD1. Another limitation of the content validation part of the study was the small sample size (n = 33), although saturation was achieved in the GD1 sample, confirming adequacy in this population. For the psychometric validation study, the target of 50 respondents was achieved, but four patients did not provide consent or failed to complete the ctGD1-PROM. Given the rarity of the disease, it was not feasible to recruit a larger sample using a single UK clinical center, and in an attempt to expand the pool of data, further psychometric validity evaluations are planned for patients with GD1 resident in Israel. However, evaluation in other, more diverse populations of patients with GD1, with varying patient characteristics and from other geographic regions, e.g. Eastern Europe, Latin America, Japan, China, India, and Africa, is needed to validate the GD-PROM in other populations, cultures, and languages.

In conclusion, both the routine monitoring and clinical trial versions of the GD1-PROM represent important steps forward towards the development of PROMs for researchers measuring the impact of GD and its respective treatment. Further validation in different populations will inform the appropriateness of the ctGD1-PROM for capturing the impact of GD on HRQoL and as a fit-for-purpose measure that meets regulatory requirements for clinical trial use.

## Supplementary Information

**Additional file 1.** Patient reported outcome measure (rmGD1-PROM).

## Data Availability

Data supporting the conclusions of this article are available on reasonable request.
