# Supplementary material for: Development and validation of Gaucher disease type 1 (GD1)-specific patient-reported outcome measures (PROMs) for clinical monitoring and for clinical trials
Source: Orphanet J Rare Dis. 2022 Jan 6;17:9. doi: 10.1186/s13023-021-02163-y (PMC8734239; doi:10.1186/s13023-021-02163-y)
Supplement: Supplementary file 1 — Additional file 1. Patient reported outcome measure (rmGD1-PROM). [file 13023_2021_2163_MOESM1_ESM.pdf]

Clinical Practice/ Routine Monitoring Version Type 1 Gaucher Disease Patient Reported Outcome Measure (rmGD1-PROM)

PART 1: Please complete each of the questions by putting a checkmark (✓) in the box which matches your best answer

	None of the time	A little of the time	Some of the time	Most of the time	All of the time	Not applicable or prefer not to say
1 Over the past month, my Gaucher disease has restricted my education/job	☐	☐	☐	☐	☐	☐
2 Over the past month, my Gaucher disease has restricted my activities with friends	☐	☐	☐	☐	☐	☐
3 Over the past month, my Gaucher disease has restricted my ability to have intimate relationships with my spouse/partner	☐	☐	☐	☐	☐	☐
4 Over the past month, my Gaucher disease has restricted my ability to take part in hobbies and leisure activities	☐	☐	☐	☐	☐	☐
5 Over the past month, I have been concerned that I am an emotional burden to others because of my Gaucher disease	☐	☐	☐	☐	☐	☐
6 Because of my Gaucher disease, I am concerned I will be at risk of bone disease	☐	☐	☐	☐	☐	☐
7 Because of my Gaucher disease, I am concerned I will be at risk of cancers	☐	☐	☐	☐	☐	☐
8 Because of my Gaucher disease, I am concerned I will be at risk of Parkinson's disease	☐	☐	☐	☐	☐	☐
9 Because of my Gaucher disease, I am concerned I will be a financial burden	☐	☐	☐	☐	☐	☐

Copyright ©2020 Takeda Pharmaceuticals International AG based on an original work created by Dr. Elstein. Published under Creative Commons License BY ND

	None of the time	A little of the time	Some of the time	Most of the time	All of the time	Not applicable or prefer not to say
10 I am concerned I will not get the best therapy because of budget issues	☐	☐	☐	☐	☐	☐
11 I am concerned I may not have an expert physician for advice in the future	☐	☐	☐	☐	☐	☐
12 My non-Gaucher problems are more concerning than the Gaucher concerns	☐	☐	☐	☐	☐	☐

	Strongly agree	Agree	Neither agree nor disagree	Most of the time	Disagree	Strongly disagree
13 Over the past month, my health in general has improved because of my Gaucher-specific medication	☐	☐	☐	☐	☐	☐
14 Over the past month, all of my medical concerns have been Gaucher-related	☐	☐	☐	☐	☐	☐
15 Over the past month, my current medication has treated my Gaucher-specific concerns	☐	☐	☐	☐	☐	☐

Copyright ©2020 Takeda Pharmaceuticals International AG based on an original work created by Dr. Elstein. Published under Creative Commons License BY ND

PART 2: Thinking about your Gaucher disease over the past week, please circle the number that is right for you

1. Over the past week, how **dependent** on others have you been because of your Gaucher disease?

Totally independent										Totally dependent
0	1	2	3	4	5	6	7	8	9	10

2. Over the past week, how **visibly big or swollen has your abdomen** looked because of your Gaucher disease?

Not big or swollen										Very big or swollen
0	1	2	3	4	5	6	7	8	9	10

3. Over the past week, how **fatigued** have you been because of your Gaucher disease?

Not at all fatigued										Severely fatigued
0	1	2	3	4	5	6	7	8	9	10

4. Over the past week, how **physically weak** have you been feeling because of your Gaucher disease?

No physical weakness										Severe physical weakness
0	1	2	3	4	5	6	7	8	9	10

5. Over the past week, how severe has your **bone pain** been because of your Gaucher disease?

No bone pain										Severe bone pain
0	1	2	3	4	5	6	7	8	9	10

Copyright ©2020 Takeda Pharmaceuticals International AG based on an original work created by Dr. Elstein. Published under Creative Commons License BY ND

6. Over the past week, how **depressed** have you been because of your Gaucher disease?

Not at all depressed										Severely depressed
0	1	2	3	4	5	6	7	8	9	10

7. Over the past week, how **worried** have you been because of your Gaucher disease?

Not at all worried										Very worried
0	1	2	3	4	5	6	7	8	9	10

8. Over the past week, how have you felt about your **future** with Gaucher disease?

Very optimistic										Very pessimistic
0	1	2	3	4	5	6	7	8	9	10

9. Over the past month, how **satisfied** have you been with your Gaucher medical treatment?

Very Satisfied										Very dissatisfied
0	1	2	3	4	5	6	7	8	9	10

Thank you for completing this form

Copyright ©2020 Takeda Pharmaceuticals International AG based on an original work created by Dr. Elstein. Published under Creative Commons License BY ND
